# From Phenomes to Genes: Phenotype-based Strategies in Rodents for Research on the Neurobiological and Genetic Bases of Psychiatric-relevant Traits

**DOI:** 10.2174/1570159X2109230518164435

**Published:** 2023-07-10

**Authors:** Alberto Fernández-Teruel

**Affiliations:** 1 Medical Psychology Unit, Department of Psychiatry and Forensic Medicine & Institute of Neurosciences, School of Medicine, Autonomous University of Barcelona, 08193-Bellaterra, Barcelona, Spain

Genetically-engineered mice or rats, *e.g*., knockout or transgenic animals, constitute experimental strategies that allow modelling psychiatric-relevant features (or traits) from an etiology-focused (bottom-up) perspective. However, one of the limitations of these approaches stems from their single-mechanism approach, which usually disregards many other facets or processes (neurobiological, genetic, environmental, neurodevelopmental, epigenetic) that may be relevant to the target phenotype/disease. This is especially true when dealing with the complexity of multicausal and polygenic psychiatric traits or syndromes.

Phenotype-based strategies in rodents, instead, constitute approaches more similar to human genome-wide association studies (GWASs). These phenotype-based (or top-down) approaches in rats and mice take advantage of the observation/measure of phenotypes that are known to be relevant to model some psychiatric-relevant trait (*e.g*., anxiety, working memory, sensorimotor gating, locomotor activity, impulsivity) and then investigate their neurobiological and/or genetic underpinnings. For example, some of these top-down preclinical models select or stratify groups of animals from a heterogeneous (outbred) population on the basis of their extreme scores in a behavioral trait (or behavioral response/s) ideally related to the target human phenotype/s or symptoms to be modelled. In other cases, large populations of phenotyped rats or mice are used for “quantitative trait loci” and “quantitative trait gene” studies. Other such models use bidirectional selective breeding (of rats or mice) for a given phenotype and then systematically evaluate the underlying neurobiological/genetic mechanisms and associated traits.

Human GWASs show that psychiatric disorders, psychiatric symptoms, and psychiatric-relevant traits are related to multiple genes (hundreds of them, in many cases) and interactions among them, as well as with the environment/ontogeny. In this regard, the above-mentioned phenotype-based approaches in rodents resemble human GWASs in that they allow to explore multiple genetic mechanisms and gene-environment interactions in intact (*i.e*., non-genetically manipulated) subjects.

The present thematic issue of Current Neuropharmacology aimed at collecting reviews representing (or summarizing) the field of rodent phenotype-based studies, which have explored the genetic and neurobiological mechanisms associated with psychiatric-relevant traits.

The review by Ramos and colleagues summarizes the usefulness of the Quantitative Trait Locus (QTL) analysis approach which, along the past 25 years, has led to advances in the genetic mapping of complex neurobehavioral traits in rodents and the identification of candidate Quantitative Trait Genes (QTG) influencing these traits. As illustrated by the authors, following such a strategy, progress has been made regarding the characterization of QTL (*i.e*., chromosomal intervals) associated with emotionality, anxiety, and other psychiatric-relevant traits, and even some QTG influencing these traits. Notably, similar to Genome Wide Association Studies (GWAS) in humans, QTL studies in rodents indicate that those complex neurobehavioral traits are influenced by multiple genes, non-coding/regulatory regions, and environmental factors. Derived from their QTL studies using crosses of the SHR and LEW rat strains and the SLA16 congenic strain, Ramos and co-workers identified a genomic region influencing emotionality/anxiety in rats, named *Anxrr16*, which contains numerous genes that might be candidate QTG for these (and related) traits. They propose several strong candidate QTG that, according to rodent (and even human) research, are involved in neural and behavioral processes (neurotransmission, emotionality/anxiety, stress, learning/memory, and others) related to the above traits. Thus, the authors propose as candidate QTG the *Npy* gene (which encodes for the NPY neuropeptide), *Crhr2* gene (which encodes for the corticotropin-releasing hormone, CRH, receptor 2), *Tacr1* gene (which encodes for the neurokinin-1 receptor, NK1R, whose highest affinity ligand is substance P), *Oxtr* gene (which encodes for the oxytocin, OXT- receptor), the *il17ra*, *il17re* and *il17rc* genes (which encode transmembrane proteins acting as interleukin-17, IL17, A, E and C receptors, IL17RA, IL17RE, and IL17RC-, respectively), and the *Grin2b* gene (which encodes for the NR2B/GluN2B subunit of glutamate NMDA receptors). Interestingly, in other rat strains differing in anxiety levels (*i.e*., selectively bred rats or outbred rats stratified for extremely divergent anxiety levels), other authors have reported differential gene expression of CRH-, OXT- and TACR-related genes, as well as of *Grin2b* and genes related to cytokine production [1-3]. These findings seem to lend further support to those reported by Ramos and colleagues in the present thematic issue.

Also, in relation to anxiety (and related disorders), Lages and colleagues present a full review of the behavioral, physiological, neuro-endocrine, and pharmacological-related findings obtained with the Carioca High- and Low-conditioned Freezing (CHF and CLF, respectively) rat lines, derived through selective bidirectional breeding for their extreme defensive responses (*i.e*., freezing) to contextual conditioned fear. The authors make a systematic comparison of the main behavioral and neurobiological findings obtained with CHF rats with those from other well-characterized selectively-bred high anxious lines/strains, such as the RLA (Roman Low Avoidance) and HAB (High Anxiety Behavior) rats. The authors propose that neurochemical and functional differences in the amygdala, hippocampus, cingulate cortex, limbic cortices, and the role of serotoninergic mediation give support to the enhanced fear- and anxiety-related responses seen in the CHF rat line, which fulfills criteria to be considered a genetically-based model of generalized anxiety disorder. Importantly, also, the CLF rat line exhibits impulsive/hyperactivity behavior (similarly to what is observed in RHA-Roman High Avoidance rats), which collectively supports the relevance of the Carioca (CHF and CLF) rat lines for preclinical neurogenetic/neurobiological research of psychiatric-relevant traits.

Redei and colleagues show that molecular/genetic markers of disease found in the Wistar Kyoto (WKY) rat model have demonstrated their translational utility to human depression. In their exhaustive review, they present compelling evidence that the WKY rat constitutes a model of human depression, presenting the crucial phenotypes of passive coping and stress hyper-reactivity, coupled with other major depression-related hallmarks, such as alterations of circadian rhythms, the hypothalamic-pituitary-adrenal (HPA) and hypothalamic-pituitary-thyroid (HPT) axes, as well as glucose metabolism. In their quantitative genetic (QTL) analyses, using reciprocal F2 generations of WKY and F344 rats, the authors found dozens of QTL influencing depression-relevant behaviors in the forced swimming test (FST), the open field test, and the defensive burying test, as well as QTL for regulation of the HPA and HPT axes and metabolic responses to stress (*e.g*., QTL on rat chromosomes 2, 3, 5, 6, among many others). The authors carried out a bidirectional selective breeding study, selecting WKY rats with “more” (WMI) or “less” (WLI) immobility in the FST, leading to high stress-reactive and low stress-reactive rats, respectively. Quantitative genetic analysis and genetic sequencing of these WKY substrains allowed finding several QTG, such as *Asic2*, *Aif1*, *Cacna1c, Cop1*, *Micb*, *Msra*, *Shank2*, and *Wnt3*, which have well-defined biological functions relevant to depression. Thus, Redei and colleagues report and summarize compelling genetic evidence further supporting the validity of the WKY as a genetic rat model of depression, which exhibits great translational value for studying human depression and testing novel therapeutics.

Differences in HPA axis function and its response to differential stressful situations are often found among rat strains that are either unselected or selected by traits not directly related to the HPA axis. In their review, Armario and colleagues extensively discuss the differences in HPA function between Lewis and Fischer rats (also including other rat strains in some comparisons, such as Brown Norway, SHR, WKY and Sprague Dawley rats) as a reference model for how to investigate strain-related differences in the HPA axis. In addition, the authors summarize and discuss the differences in HPA function between some classical rat strain pairs, such as the high- and low-anxiety selected rats, the Roman high- and low-avoidance strains/lines, WKY and SHR rats, WKY *vs*. other rat strains, the Hatano high- and low-avoidance rats, the Syracuse high and low-avoidance lines, and the Flinders Sensitive and Flinders Resistant rat lines. Importantly, it is discussed, in light of between-strain differences in HPA axis and anxiety/coping behaviors, whether these behavioral traits (or states) are related to HPA function. The authors conclude that the findings reported to date give a complex picture and do not support a simple relationship between both aspects. Importantly, the authors extensively discuss a series of methodological issues related to HPA axis studies, as well as their implications, and provide a recommended guide to better interpret experimental data about the HPA axis. Given that HPA function is considered to be critical for coping and adaptation to stress, and it has been associated with a number of psychiatric diseases (*e.g*., anxiety, depression), the review by Armario and coworkers and the methodological issues addressed constitute an important contribution to the field.

Deficits of inhibitory control are at the core of impulsivity- and compulsivity-related disorders, such as obsessive-compulsive disorder (OCD), schizophrenia, addiction, and attention-deficit hyperactivity disorder, among others. Hence, preclinical neurobiological research on inhibitory control alterations has an important potential translatability to human psychiatric conditions. Martín-González and colleagues present a review on the neurobiological bases of compulsivity using the “Schedule-induced Polydipsia (SIP)” model, in which rodents develop adjunctive, excessive and persistent drinking behavior under different intermittent food-reinforcement schedules. They have developed a model of High-compulsive (HD; high drinker) *vs*. Low-compulsive (LD; low-drinker) rats by stratifying Wistar rats according to their very high (HD) or low (LD) drinking responses in the SIP procedure. Using the HD *vs*. LD rat model, the authors provide pharmacological and neurochemical evidence suggesting that putative genetic alterations of this preclinical model of compulsivity might be found in relation to dopamine D1 and D2 receptors, serotonin 5-HT2A receptors and glutamatergic transmission. Research on the HD *vs*. LD rats seems likely to have significant translational potential to increase our knowledge on the neurobiological/neurogenetic bases of compulsive behavior, characteristic of OCD and other psychiatric disorders.

Finally, Oliveras and colleagues review most of the behavioral, neurobiological and genetic research carried out with the main rat lines/strains used as models of schizophrenia-relevant features. These six genetically-derived (through bidirectional selective breeding in most cases) and phenotype-based rat models are the APO-SUS (*vs*. APO-UNSUS), Low-PPI (*vs*. High-PPI), Wisket (*vs*. Wistar controls), RHA (*vs*. RLA), Brattleboro (*vs*. Long-Evans controls) and SHR (*vs*. WKY) rat lines/strains. Remarkably, all these six selectively-bred rat models share sensorimotor gating deficits (*e.g*., deficits in prepulse inhibition, PPI, which are also characteristic of schizophrenia), and most of them exhibit increased novelty-induced activity and deficits in latent inhibition, social behavior, and cognitive flexibility (phenotypes that are also found in patients with schizophrenia). Regarding neurochemical processes, alterations of dopamine transmission or dopamine (D1, D2) receptors are found in most of the models, whereas prefrontal cortex (PFC) dysfunction (characteristic of schizophrenia) is observed in four out of the six rat lines/strains. Regarding genetic/molecular findings with these six models, it is worth highlighting that some of them exhibit altered expression of schizophrenia-related genes (mainly in the PFC), such as myelin-related genes, oxytocin-related genes, increased *Nrg1* (neuregulin-1) expression, increased *Homer1* expression, enhanced *BDNF* expression, and alterations of dopamine, serotonin and glutamate receptors as well as of presynaptic markers of the SNARE complex. Although at least four out of the six models have shown sensitivity to pro-psychotic and/or antipsychotic drug treatments, the authors emphasize that there is still a long way to go regarding the pharmacological characterization of these models, so systematic and extensive drug treatment studies are necessary to see whether they present good predictive validity and selectivity (*e.g*., absence of false positives or false negatives). Meanwhile, Oliveras and colleagues suggest that future research oriented along the Research Domain Criteria (RDoC) framework, *i.e*., research strongly based on genetic and neuroscientific grounds, might lead to advances in understanding the neurogenetic and circuit mechanisms related to specific behavioral phenotypes/traits (*e.g*., cognitive flexibility, working memory, latent inhibition, sensorimotor gating, social withdrawal, *etc*.), which in turn may be important for symptoms that are currently classified as relevant for schizophrenia.

Collectively, the reviews in this Thematic Issue of Current Neuropharmacology constitute examples of how some outstanding phenotype-based (top-down) research strategies have led to remarkable progress in our knowledge on psychological, neurobiological and (neuro-) genetic mechanisms associated with the domains of emotionality, anxiety, depression, stress sensitivity and responses, impulsivity/compulsivity, obsessive-compulsive disorder, and schizophrenia-related traits. There are many more examples of this research strategy, which further support the usefulness and translational potential of this approach. For example, in a pioneering quantitative genetic study on behavioral traits, Flint *et al.* [4] identified several QTL for anxiety in a large sample of F2 hybrid mice (derived from crossing high-anxious and low-anxious mice). This research was followed by another in which the authors fine-mapped QTL for anxiety in genetically heterogeneous mice [5], and from this QTL study, the authors identified a QTG for anxiety, the *Rgs2* gene [6], which was validated in anxious human samples (*i.e*., it was predictive of anxiety in humans) [7].

Along similar lines, using F2 rats derived from crossing the RHA (low anxious) and RLA (high anxious) strains, a pleiotropic QTL for conditioned anxiety was found in chromosome 5 [8]. This was followed by fine-mapping of that QTL in genetically-heterogeneous (HS) rats [9]. Further, QTL fine-mapping studies in a very large sample of HS rats led to the identification of the Catenin-delta 2 (*Ctnnd2*) gene in chromosome 2 as a QTG (quantitative trait gene) for conditioned anxiety [10-12]. *Ctnnd2* was also validated in knockout and transgenic mice [13, 14], as well as in a human psychiatric sample [15]. Another example is the *Mpdz* gene, which was identified as a QTG for alcohol dependence in mice after several QTL studies [16-18].

The above are just some representative and relevant examples of how from a phenotype-based approach, starting with QTL studies on crosses of mice or rat strains showing extreme scores in a given phenotype or trait, this research strategy has produced outstanding findings on the genetic bases of complex behavioral traits. Similar approaches have been (and are being) used to explore QTL (and QTG) of other psychiatric-relevant traits in rodents, such as sensorimotor gating [19-21], depression-related phenotypes [22, 23], drug-addiction traits [24, 25], and many others. It is important to highlight that these research strategies always need to start from a good characterization and understanding of the phenotypes, which has been the starting point and the scope of the present Thematic Issue.
